# The role of financial stress in mental health changes during COVID-19

**DOI:** 10.1038/s44184-022-00016-5

**Published:** 2022-10-14

**Authors:** Olaf Simonse, Wilco W. Van Dijk, Lotte F. Van Dillen, Eric Van Dijk

**Affiliations:** 1 Faculty of Social and Behavioural Sciences of Leiden University, Leiden, Netherlands; 2 Knowledge Center Psychology and Economic Behavior, Leiden, Netherlands; 3 Ministry of Finance, the Hague, Netherlands

**Keywords:** Psychology, Human behaviour, Psychology, Human behaviour

## Abstract

Using longitudinal data before and during the first six months of the COVID-19 pandemic for a representative sample of Dutch households, we examined the role of financial stress, defined as the subjective experience of lacking financial resources to cope with demands, in mental health changes. Also, we examined financial stress and mental health relations with households’ income, savings, and debts. The data revealed that average mental health did not change during the first six months of the pandemic but showed considerable underlying heterogeneity. Results showed that financial stress changes significantly explained this heterogeneity. Increases in financial stress predicted decreases in mental health, whereas decreases in financial stress predicted increases in mental health. While income did not explain financial stress changes, fewer savings and more debts were related to increased financial stress, which was, in turn, negatively related to mental health. We discuss the implications of our findings for mental health care and financial security policy and provide suggestions for future research.

## Introduction

On 11 March 2020, the World Health Organization (WHO) declared COVID-19 a pandemic^1^. Health authorities quickly realized that the pandemic posed a physical and mental health threat. On 18 March 2020, the WHO wrote, “this time of crisis is generating stress throughout the population” and called upon policymakers, health care professionals, and the general population to “support mental and psychosocial wellbeing in different target groups during the outbreak.” Based on experience with previous pandemics, such as the Spanish flu (1918–1920), the Asiatic flu (1956–1957), the Severe Acute Respiratory Syndrome (SARS, 2002–2003), the “Swine” flu (2009), and Ebola (2013–2014), researchers proposed that the mental health consequences of the COVID-19 crisis were likely to be present for a long time and peak later than the actual pandemic^2–4^. They called upon the research community to study the mental health effects of COVID-19.

Studies of mental health development during the pandemic have found mixed results. Some studies observed negative mental health outcomes^5–10^, whereas others reported positive aspects of the pandemic^11,12^ or found no evidence of changes in mental health outcomes during the pandemic^11,13,14^. Robinson et al.^8^ observed a high degree of unexplained heterogeneity in mental health responses to COVID-19. The most reported symptoms have been post-traumatic stress^5–7^, depression^6–8,10^, and anxiety^5,10,12,15–17^. Other reported symptoms include insomnia^5,18^ and loneliness^19^.

Scholars have proposed three potential pathways by which the pandemic may affect mental health: the disease itself, the quarantine measures, and the economic consequences of the pandemic. As for the first pathway, the disease (threat) may directly affect mental health. People may fear that they or their significant others may be infected^4,15^. Those who catch the disease may suffer post-infection consequences, such as fatigue and pain^20^ and fear of being a burden to those around them^4^. The second pathway acknowledges that measures to contain the disease, such as quarantine and social distancing, may affect mental health by reducing opportunities for physical and mental health activities, such as recreational activities and routines^15,21–23^. The third pathway assumes that mental health may suffer from the economic consequences of the pandemic^15,22^. In the current study, we focus on this economic pathway, particularly the potential role of financial stress in explaining changes in mental health.

Research suggests that, as a consequence of these three pathways, socio-economically disadvantaged groups are more vulnerable to mental health problems during the pandemic^11,12,19–21,24–27^. First, low socio-economic status is associated with a higher chance of COVID-19 infection, resulting in higher mental distress^28,29^. Second, low-income jobs are less likely to be executed from home, so they are most affected by the lockdown and social distancing measures^30^. This may also result in increased role conflicts having to combine work and family obligations^30,31^. Third, socio-economically disadvantaged and financially vulnerable groups are more likely to suffer the pandemic’s economic consequences. They are likelier to work in sectors that suffered the most from COVID-19, such as restaurants, travel, entertainment, and certain retail branches. Also, workers most likely to be affected by unemployment are less educated and have fewer financial resources. An empirical study among people across the European Union in the first six months of the pandemic showed high job insecurity among those with temporary contracts. Also, the unemployed had difficulty making ends meet, and people with low job insecurity had considerable mental health issues^32^. A cross-sectional study among 1441 US citizens in the first two months of the pandemic showed that financial stressors and low assets were associated with higher odds of depression^33^. Financial stressors were defined as losing a job, a household member losing a job, having financial problems, and having difficulty paying rent. Assets included social assets (education and marital status), physical assets (homeownership), and financial assets (household income and household savings). Despite considerable support for a negative relationship between socio-economic status and mental health outcomes, some studies do not find such a relationship^5,34,35^. For example, Pijpker et al. found no differences in mental health between low and high socio-economic status respondents in a sample of the Dutch population^36^.

Entrepreneurs, particularly self-employed, are another group that suffered from the economic consequences of the pandemic. They experienced a higher loss of working hours than others during the pandemic^37,38^. Several studies indicate that self-employed people are susceptible to mental health problems due to the pandemic’s economic consequences of the pandemic^26,39,40^. This finding should be treated with caution; a recent systematic review of studies comparing mental disorders in the self-employed versus employees found evidence of a link between self-employment and increased risk of mental illness^41^.

Research on the relationship between the economic situation of households and mental and physical health has a long history. In the 1980s, Rose and Marmot followed more than 17,000 municipal officials in London. Their well-known Whitehall Studies showed that lower-paid civil servants were more likely to develop cardiovascular disease than their colleagues with higher positions^42^. Since then, studies have shown the relationship between poverty and many physical and mental conditions, such as diabetes, cancer, chronic lung disease, schizophrenia, depression, substance use, and anxiety disorders^43–45^.

When in financially challenging circumstances, such as low income or debt, people can experience financial stress^46^. Financial stress is a psychological concept characterized by the subjective experience of lacking financial resources to cope with demands^47^. In the current study, we conceptualize financial stress as the combination of two stress appraisals (money shortage and lack of control) and two stress responses (worrying about money and short-term focus)^48,49^.

There is evidence that financial stress mediates the relationship between poverty and health^50^. Poor households often have fewer resources (for example, financial buffers in savings and social support) to deal with life events. This lack of resources may result in stress and health problems^51,52^. Debt is also associated with stress and mental health problems^53^. Income fluctuations cause uncertainty and, therefore, stress^54,55^. Having savings to deal with setbacks reduces stress and increases financial wellbeing^56^.

Although the evidence is mixed, most studies have found that mental health declined during the COVID-19 pandemic. Research also indicated a high degree of unexplained heterogeneity in mental health changes. Many studies on COVID-19 and mental health cannot adequately examine these changes because these studies have cross-sectional designs. When studies used longitudinal designs, data collection (understandably) started only after the pandemic outbreak. The current study examined mental health changes by including data collected before and after the pandemic outbreak; this was possible by connecting long-running data on mental health to an ongoing data collection on financial stress^49^. The current study specifically focused on how (changes in) financial stress might explain these mental health changes.

Moreover, we examined how households’ financial situation before COVID-19 and their income development during COVID-19 explained financial stress. Having savings may protect against financial stress because savings can absorb income loss or unexpected expenditures. Especially in economically uncertain times, lacking sufficient savings may result in feelings of not being in control of one’s financial situation and in worries about being unable to meet financial obligations. Thus, low levels of savings may result in increased financial stress. Similarly, having debts in economically uncertain times may trigger worries about being unable to repay them because of the anticipation of future income drops. Also, having debts may increase feelings of dependency on others^57^. Thus, having debts in economically uncertain times such as COVID-19 may increase financial stress. Also, it stands to reason that income and financial stress are dynamically related: income drops are likely associated with increasing financial stress, given that a large portion of households’ expenditures (e.g., rent, insurance, and utilities) is fixed. Finally, households’ income level is likely to be negatively associated with financial stress. Low-income households are more vulnerable to becoming unemployed. Moreover, low-income households may have fewer opportunities to cut spending. We tested three hypotheses:

1. Increases in financial stress during COVID-19 positively relate to decreased mental health, whereas decreases in financial stress relate to increased mental health.
2. Falling incomes during COVID-19 and low incomes, low savings, and high debts before COVID-19 relate to increases in financial stress during COVID-19.
3. Changes in financial stress during COVID-19 mediate the association between financial vulnerability (in terms of income drops, low incomes, low savings, and high debts) and mental health changes.

## Methods

### Data and variables

We used data from the Longitudinal Internet Studies on Social Sciences (LISS) panel (initial *N* = 1114). The LISS panel consists of a representative sample of approximately 5,000 households drawn by the Central Bureau of Statistics of the Netherlands^58^. Respondents fill in monthly questionnaires on various topics, such as health, family, work, personality, and economic situation. To ensure that vulnerable households can participate, they are supplied with a laptop and internet connection if necessary. The rich dataset enabled us to examine the relationship between developments in households’ economic situation, financial stress, and mental health. We used three measurements to compare the situations before and during COVID-19: April – November 2018 (*t* = 0), December 2019 – March 2020 (*t* = 1), and December 2020 – March 2021 (*t* = 2).

The methods were performed in accordance with relevant guidelines and regulations and approved by CentERData. The current study used secondary data provided by CentERData. Informed consent was obtained from all participants by CentERData. Before participating in the LISS panel, participants must consent to CentERData to save their responses and make them available for scientific, policy, and social research.

#### Mental health

The literature suggests that the most prevalent mental health problems related to COVID-19 are anxiety and mood disorders. To assess mental health, we, therefore, used the Mental Health Index (MHI-5), a brief and reliable measure of mental health with good validity for anxiety and mood disorders^59^, and a subset of the validated SF-36 Health Survey^60^ (Cronbach’s *α* = 0.87). MHI-5 asks respondents how often they felt nervous, down, calm, depressed, and happy in recent weeks. Respondents’ scores on each item ranged from 1 (never) to 6 (continuously). We recoded the items so that a higher score reflected better mental health. LISS’ health questionnaire measures MHI-5 every year. We used the measurements administered in November/December 2018, 2019, and 2020.

#### Explanatory variables

We used the Psychological Inventory of Financial Scarcity (PIFS) (Cronbach’s *α* = 0.93) to measure financial stress^48,49^. The PIFS assesses the subjective experience of financial stress and captures appraisals of insufficient financial resources and lack of control over one’s financial situation, responses regarding financial rumination and worry, and a short-term focus. Respondents’ scores on each item range from 1 (totally disagree) to 7 (totally agree). Higher scores indicate more financial stress. The PIFS was administered in April 2018, February 2020, and August 2020.

We included four aspects of a household’s economic situation in the analyses: income, income volatility, savings, and debts. We used monthly income data for 2018, 2019, and 2020. For savings and debts, we used the last available measurement before the outbreak of COVID-19. This measurement was held in June/July 2019 and concerned households’ financial situation at the end of 2018.

#### Income

The LISS panel measures net monthly household income in euros. We summed the net monthly household incomes for 2018, 2019, and 2020 to obtain yearly net household incomes. Since the needs of a household grow with each additional member, we corrected for household size. To consider economies of scale, we adjusted household income by dividing it by the square root of household size, in line with OECD guidance^61^. We included income at the first measurement and income changes between the three measurements as independent variables in our model.

#### Savings

Savings may serve as buffers against unexpected expenditures and income shocks. Ruberton et al. stressed the importance of liquid wealth for wellbeing^56^. We, therefore, included the amount of household liquid savings in our analyses. Respondents were asked: “What was the total balance of your banking account, savings accounts, term deposit accounts, savings bonds or savings certificates, and bank savings schemes on 31 December 2018?”. If they responded, “I don’t know,” the questionnaire asked, “To what category did the total balance (total value) belong on 31 December 2018 (positive or negative)?” and given 15 categories (less than € 50 to € 25,000 or more). We used the category midpoints to calculate savings.

#### Debts

To calculate debt amounts, we excluded mortgages and student loans from our analyses and focused on consumer credit. We argue that, for most households, having a mortgage contributes less to financial stress than other types of debt since a mortgage is not a sign of financial difficulties in most situations. Also, the home’s value usually amply compensates for the mortgage loan’s value. Student loans in the Netherlands have favorable conditions and are waivered if one has difficulties repaying them and should, therefore, also contribute less to financial stress. The survey asked respondents to indicate whether they had (a) one or more personal loans, revolving credit arrangement(s), or financing credit(s) based on a hire-purchase or installment plan, (b) a loan or credit arrangement based on a pledge, (c) overdue payments on one or more credit cards (d) money loaned from family, friends, or acquaintances, and (e) any other credits, loans or debts. Respondents indicating that they held one or more of these debts were then asked: “What was the total amount of the loans, credits, and debts that you had on 31 December 2017? This concerns the total of all the components you check-marked in the previous question.” If they responded, “I don’t know,” the questionnaire asked, “To what category did the total balance (total value) belong on 31 December 2018 (positive or negative)?” and given 14 categories (less than € 500 to € 100,000 or more). We used the category midpoints to calculate debt amounts.

#### Control variables

We used age, education level, household composition, and personality traits as control variables in our analyses. Age and education level may confound the association between income and financial stress. Furthermore, research has shown that mental health during COVID-19 may differ between households with different compositions^12,19,20,35^. We distinguished four household types: (1) no partner, no children, (2) children, no partner, (3) partner, no children, and (4) partner with children.

We considered the Big-Five personality traits (extraversion, agreeableness, openness, conscientiousness, and emotional stability)^62^ as potential confounders of the relationship between mental health and one or more independent variables. Several studies have indicated that personality traits influence saving behavior, impulse buying, debts, and financial stress. The literature provides the most support for extraversion, conscientiousness, and emotional stability as potential covariates. For example, conscientiousness is positively associated with savings and negatively with debts^63^ and financial stress. Extraversion negatively predicts debts^64^. Emotional stability shows a negative association with financial stress^48^. We, therefore, included subscales for emotional stability, conscientiousness, and extraversion (*α* = 0.77, 0.89, and 0.87, respectively) in our analyses.

We parsed out the variance between six controls (age, education level, household composition, emotional stability, conscientiousness, and extraversion) and the independent variables. This allowed us to examine the unique relationship between economic variables, financial stress, and mental health.

### Model

A linear mixed model analyzes the dynamic relationship between variables of interest within and across individuals. We were interested in how financial stress and mental health changes were related. Moreover, we wanted to establish indirect relations between income changes during COVID-19, income, savings, and debts before COVID-19, on the one hand, and mental health changes on the other. In addition, we wished to allow for individual heterogeneity in mental health. We, therefore, chose a random intercepts model, meaning that the average mental health and financial stress over the three observations may differ between individuals. At the same time, the slopes are homogeneous for the sample. We included time as an independent variable to test whether mental health and financial stress changed between measurements. Also, we added time as a moderator to our model to test whether the relationship between mental health and financial stress differed between the three measurements.

Furthermore, we did not impose any restrictions in advance on the covariance between observations at different measurement moments (unstructured covariances). We standardized the numeric variables to ease the interpretation of the parameter estimates. We estimated a mediation model to test our hypotheses, where mental health was the dependent variable, financial stress was the mediator, and income, savings, and debts were the independent variables. The following equations describe the model mathematically:

1 $${{{\mathrm{y}}}}_{{{\mathrm{t}}}} = {{{\mathrm{\alpha }}}} + {{{\mathrm{\beta x}}}}_{{{\mathrm{t}}}} + {{{\mathrm{\gamma m}}}}_{{{\mathrm{t}}}} + {{{\mathrm{\delta z}}}} + {{{\mathrm{\varepsilon }}}}_1{{{\mathrm{t}}}} + {{{\mathrm{\varepsilon }}}}_2{{{\mathrm{tm}}}}_{{{\mathrm{t}}}} + {{{\mathrm{\eta }}}}_{{{\mathrm{t}}}}$$

2 $${{{\mathrm{m}}}}_{{{\mathrm{t}}}} = {{{\mathrm{\alpha }}}}^{\prime} + {{{\mathrm{\beta }}}}^{\prime} {{{\mathrm{x}}}}_{{{\mathrm{t}}}} + {{{\mathrm{\delta }}}}^{\prime} {{{\mathrm{z}}}} + {{{\mathrm{\alpha }}}}^{\prime} + {{{\mathrm{\varepsilon }}}}^{\prime} _1{{{\mathrm{t}}}} + {{{\mathrm{\eta }}}}^{\prime} _{{{\mathrm{t}}}}$$

In these equations, *t* represents the time of the measurement (*t* = 1, 2, 3), y_t_ is a vector of length *N*  = 1114 with the dependent variable mental health at measurement *t* for each respondent. x_t_ Is a vector with the time-dependent variable income at time t. z Is a matrix with constant variables over time: the independent variables (savings and debts) and control variables (age, education level, gender, household composition, and personality traits). m_t_ is a vector with the mediator financial stress at measurement t; tm_t_ represents the interaction between time and the mediator financial stress. α and α′ Are vectors with random intercepts. β, γ, δ, ε_1_, ε_2_, β′, γ′, δ′, ε′_1_ are the regression coefficients, and η_t_ and η′_t_ are the prediction errors.

### Analyses

Our statistical analyses were designed to deal with missing values and outliers. First, many observations had missing data on one or more variables. All variables, except age and gender, had missing values; 15% were missing, and 67% of the observations had a missing value on at least one variable. Missing values on the financial stress measurements were due to attrition; the reasons for missing values on the other variables are unknown. Second, an inspection of diagnostics from the OLS regression showed many influential observations (outliers). In our analyses, we addressed these data characteristics by performing multiple imputation and choosing a robust regression method for influential observations. Because the regressions tested multiple null hypotheses, we adjusted the *p*-values as proposed by Benjamini and Yekutieli to control for false discovery rates^65,66^.

#### Multiple imputation

Deleting observations with missing values on one or more variables would leave 67% of the observations unused, resulting in inflated standard errors^67^. If the attrition is selective, the resulting estimations may be biased. Multiple imputation reduces standard errors and bias^67,68^. We selected an iterative Monte Carlo Markov Chain (MCMC) mechanism to generate imputations and used the R package *jomo* to perform the imputations^69^. MCMC assumes multivariate normality but performs well if this assumption does not hold^70^. For the imputation, we did not consider the longitudinal structure of the data. Previous research has shown that reflecting this structure in the imputation process is not needed^71^. To increase the plausibility of missingness at random, we included the control variables age and gender as auxiliary variables in the imputation process^70^. A test run with 20 imputations, using Satterthwaite’s correction for the degrees of freedom, resulted in a maximum fraction of missing information (fmi) of 0.64^72^. Based on Von Hippel’s guidance, we set the number of imputations at 101, corresponding with a 5% variation in the standard error estimates^73^. We performed the subsequent analyses with each of the 101 imputed datasets and combined the results using Rubin’s rules^74^. The parameter estimates are simply the averages over the imputations. The standard error is the square root of the within-imputation variance and the between-imputation variance.

#### Robust multivariate regression

It is well established that ordinary least squares (OLS) estimation can give highly unreliable outcomes in the presence of influential observations. OLS minimizes the sum of the squared residuals, which offers “unusual” observations an unduly large weight. We applied the *robustlmm* package in R to generate robust parameter estimates for our linear mixed-effects model^75^. This package minimizes a smoothed version of the Huber function^76^. It uses an iterative reweighing algorithm to estimate the model parameters.

To establish whether financial stress mediated the association between respondents’ economic situation and their mental health, we calculated the indirect associations using the distribution-of-the-product method proposed by MacKinnon^77,78^.

### Reporting summary

Further information on research design is available in the Nature Research Reporting Summary linked to this article.

## Results

### Descriptive statistics

Table 1 summarizes sample statistics. The initial sample contained 1114 respondents. Attrition was 25% between the first and the second measurement and 12% between the second and third measurements. Inspection of the descriptives for the three measurements reveals that – on average – those who remained in the sample had somewhat higher incomes and were slightly older than those who dropped out (note that “Age” in Table 1 represents the age at the first measurement). Financial stress, on average, was low and mental health was relatively high in all three measurements. Average financial stress was stable in the first two measurements (1.78 and 1.76, respectively) and declined somewhat in the third (1.63). Mental health remained virtually unchanged in the three measurements (4.13, 4.14, and 4.17, respectively).

**Table 1:** Descriptive statistics.

Characteristic	*t* = 0: *N* = 1114	*t* = 1: *N* = 838	*t* = 2: *N* = 736
Net income	32,688 (21,575, 46,225)	34,100 (22,800, 47,950)	34,380 (22,800, 48,068)
Age (years)	53.0 (17.8) [18.0 92.0]	54.5 (16.9) [18.0 92.0]	55.6 (16.6) [18.0 92.0]
Education level			
Primary school	65 (5.8%)	46 (5.5%)	40 (5.4%)
vmbo (intermediate secondary education)	220 (20%)	180 (22%)	160 (22%)
havo/vwo (higher secondary education)	133 (12%)	94 (11%)	80 (11%)
mbo (intermediate vocational education)	269 (24%)	208 (25%)	183 (25%)
hbo (higher vocational education)	283 (25%)	213 (25%)	190 (26%)
wo (university)	143 (13%)	96 (11%)	82 (11%)
Gender: female	613 (55%)	451 (54%)	390 (53%)
Household composition			
No partner, no children	301 (30%)	249 (30%)	218 (30%)
No partner, with children	37 (3.7%)	34 (4.1%)	27 (3.7%)
Partner, no children	381 (38%)	329 (39%)	298 (40%)
Partner, with children	293 (29%)	226 (27%)	193 (26%)
Savings	35,906 (72,592) [−8000 662,957]	38,950 (78,269) [−950 662,957]	40,726 (81,179) [−950 662,957]
Debt amount	2216 (18,110) [0 320,000]	2207 (18,624) [0 320,000]	1701 (13,924) [0 216,000]
Financial stress (1–7)	1.78 (1.03) [0.92 6.42]	1.76 (1.04) [0.92 6.42]	1.63 (0.96) [0.92 6.42]
Mental health index (1–6)	4.14 (0.85) [1.00 5.40]	4.13 (0.83) [0.60 5.40]	4.17 (0.84) [0.40 5.40]

Median (IQR); mean (SD) [minimum maximum]; *N* (%).

**Fig. 1:**
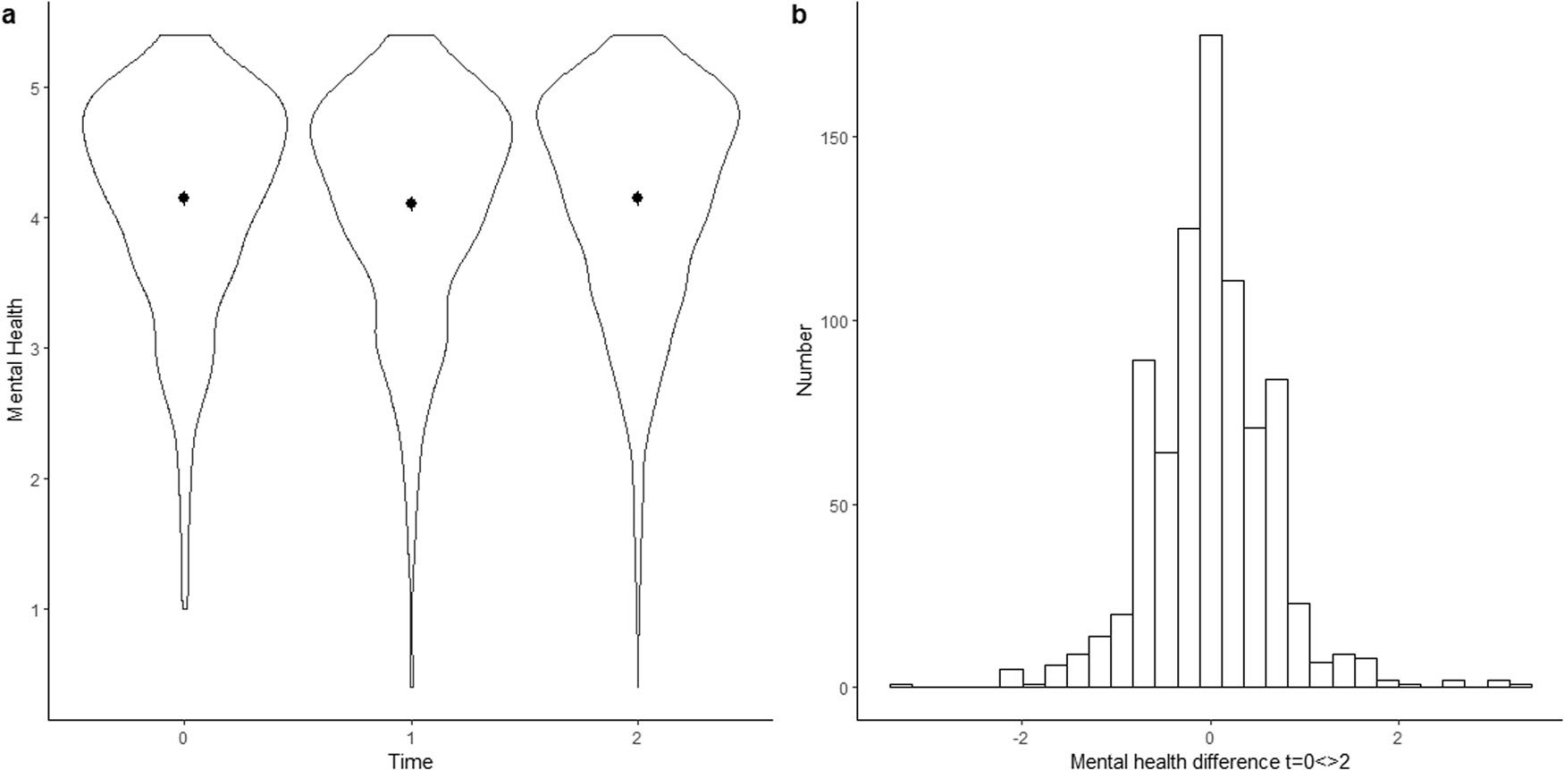
Development of mental health during COVID-19. **a** Average mental health at *t* = 0 (November/December 2018), *t* = 2 (November/December 2019), and *t* = 2 (November/December 2020); **b** Differences in mental health between *t* = 0 and *t* = 2.

**Fig. 2:**
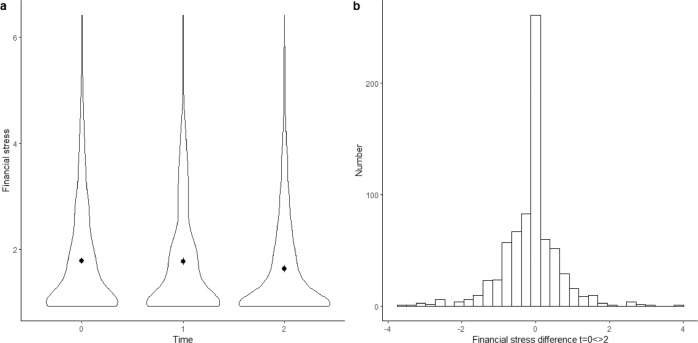
Development of financial stress during COVID-19. **a** Average mental health at *t* = 0 (April 2018), *t* = 2 (February 2020), and *t* = 2 (August 2020); **b** Differences in mental health between *t* = 0 and *t* = 2.

Figure 1 provides a graphical presentation of mental health development during COVID-19. There are no observable shifts in average mental health between November/December 2018 and November/December 2020 (see Fig. 1a). This corroborates the findings of the Dutch Social Planning Office and the Dutch Health Council^13,79^. However, we observed considerable variation in mental health changes (see Fig. 1b). For large proportions of respondents, mental health increased (39%) or decreased (40%) between the first and last measurements. For 21% of the respondents, mental health did not change. In sum, while the mean level of mental health appeared stable, we observed considerable heterogeneity among respondents. A similar pattern emerged for financial stress (see Fig. 2): on average, financial stress was stable, but there was considerable individual heterogeneity.

Supplemental Table 1 provides statistics for the three groups of respondents: those with decreased, unchanged, and increased mental health. On average, those with unchanged mental health had higher adjusted incomes than those with decreased or increased mental health. Adjusted incomes increased in all three groups, but the adjusted income increase was the lowest in the group with decreased mental health. In the group with decreased mental health, median savings were lower (€ 36,667) than in the group with unchanged mental health (€ 48,364) but somewhat higher than in the group with increased mental health (€ 33,137). The median debt amount was the highest in the group with decreased mental health (€ 3135), compared to the group with unchanged mental health (€ 458) and increased mental health (€ 1947). Financial stress decreased in all three groups, but there was more variability in the group with decreased mental health.

The correlations between mental health at the three measurements were around 0.7 (Table 2). For financial stress, correlations between the three measurements were between 0.6 and 0.8 (Table 3). We can interpret these correlations as mental health and financial stress parts that are more or less constant and determined by stable intra-individual factors such as demographic variables and personality traits. Although these autocorrelations are moderate to high, they are not perfect. These imperfect correlations confirm the view that there are dynamics in the two variables, which stable factors do not explain.

**Table 2:** Pearson’s correlations (two-sided) between the three mental health measurements.

Mental health	*t* = 0	*t* = 2
*t* = 1	0.73***	–
*t* = 2	0.71***	0.72***

Sig: * = <0.05. ** = <0.005, *** = <0.0005.

**Table 3:** Pearson’s Correlations (two-sided) between the three financial stress measurements.

Financial stress	*t* = 0	*t* = 1
*t* = 1	0.70***	–
*t* = 2	0.69***	0.81***

Sig: * = <0.05. ** = <0.005, *** = <0.0005.

### Regression results

Regression results partly confirmed our three hypotheses. Changes in financial stress predicted changes in mental health; in line with hypothesis 1, increases in financial stress were positively related to decreases in mental health (*β* = −0.119, −*t*(667) = 5.25, *p* < 0.001) (Table 4). Increases in financial stress, in turn, were predicted by low savings (*β* = 0.141, *t*(122) = −3.53, *p* = 0.005) and high debt levels (*β* = 0.912, *t*(240) = 3.41, *p* = 0.008) before COVID-19, in line with hypothesis 2 (Table 5). Also, changes in financial stress mediated the association between savings and debts on the one hand and changes in mental health on the other, in line with hypothesis 3 (95% CI [.00662, 0292]). However, we did not find support for an association between savings (*β* = 0.081, *t*(161) = 0.272, *p* = 0.125) and debts (*β* = 0.021, *t*(316) = 0.95, *p* = 1) on the one hand and mental health on the other. We found no support for income just before the pandemic (*β* = 0.098, *t*(232) = 2.08, *p* = 0.416) and income changes during the pandemic (*β* = −0.084, *t*(136)) = 0.994, *p* = 0.416) as explanatory variables for financial stress and mental health changes. Finally, we found no support for an indirect association between income and mental health (95% CI [−0.04, 0003]).

**Table 4:** Regression results for Eq. (1).

Dependent variable: mental health	Estimate	SE	t	df	p	Sig
Intercept	−0.160	0.095	−1.69	551	0.881	
Financial stress	−0.119	0.023	−5.25	677	<0.001	***
Adjusted income (*t* = 0)	0.098	0.047	2.08	232	0.416	
Δ Adjusted income	−0.084	0.055	−1.53	136	0.994	
Savings	0.081	0.030	2.72	161	0.125	
Debts	0.021	0.022	0.95	316	1	
*t* = 0	−0.011	0.022	−0.50	913	1	
*t* = 1	−0.005	0.023	−0.21	622	1	
Financial stress* (*t* = 0)	0.019	0.024	0.77	628	1	
Financial stress* (*t* = 1)	0.020	0.026	0.75	392	1	
Age	0.029	0.025	1.16	491	1	
Gender (F)	−0.011	0.020	−0.54	776	1	
Education level: 2	0.151	0.096	1.57	640	0.994	
Education level: 3	0.022	0.105	0.21	584	1	
Education level: 4	0.110	0.096	1.14	565	1	
Education level: 5	0.103	0.095	1.09	598	1	
Education level: 6	−0.017	0.108	−0.15	497	1	
Household: no partner, with children	0.158	0.111	1.43	666	1	
Household: partner, no children	0.145	0.051	2.86	598	0.095	
Household: partner, with children	0.126	0.056	2.23	575	0.374	
Conscientiousness	0.048	0.023	2.09	442	0.416	
Emotional stability	−0.501	0.024	−21.26	521	<0.001	***
Extraversion	−0.064	0.022	−2.89	488	0.095	

*SE* standard error, *p* adjusted *p*-value (two-sided *t*-test, adjusted with Benjamini and Yekutieli correction).

Sig: * = <0.05. ** = <0.005, *** = <0.0005.

**Table 5:** Regression results of Eq. (2).

Dependent variable: financial stress	Estimate	SE	*t*	df	*p*	Sig
Intercept	0.4653	0.112	4.16	504	<0.001	***
Adjusted income (*t* = 0)	0.1428	0.087	1.65	98	0.462	
Δ Adjusted income	−0.1753	0.072	−2.42	95	0.089	
Savings	−0.1414	0.040	−3.53	122	0.005	**
Debts	0.0912	0.028	3.31	240	0.008	**
*t* = 0	0.0284	0.021	1.36	764	0.737	
*t* = 1	0.0552	0.022	2.46	500	0.078	
Age	−0.0928	0.030	−3.11	476	0.013	*
Gender (F)	−0.0667	0.024	−2.73	637	0.039	*
Education level: 2	−0.4870	0.117	−4.17	514	<0.001	***
Education level: 3	−0.4442	0.126	−3.52	511	0.004	**
Education level: 4	−0.4636	0.113	−4.09	550	<0.001	***
Education level: 5	−0.5259	0.113	−4.65	543	<0.001	***
Education level: 6	−0.6026	0.126	−4.77	505	<0.001	***
Household: no partner, with children	0.1417	0.134	1.06	580	1	
Household: partner, no children	−0.1274	0.061	−2.09	538	0.178	
Household: partner, with children	0.0288	0.067	0.43	555	1	
Conscientiousness	−0.1188	0.027	−4.37	415	<0.001	***
Emotional stability	−0.2403	0.027	8.84	517	<0.001	***
Extraversion	0.0076	0.026	0.30	537	1	

*SE* standard error, *p* adjusted *p*-value (two-sided *t*-test, adjusted with Benjamini and Yekutieli correction).

Sig: * = <0.05. ** = <0.005, *** = <0.0005.

We did not find an association between time and mental health (*β* = −0.011, *t*(913) = 0.50, *p* = 1 and *β* = 0.005, *t*(622) = −0.21, *p* = 1 for *t* = 0 and *t* = 1, respectively). This corroborates our earlier observation that, on average, mental health did not change during the assessed period before and during the COVID-19 pandemic. Moreover, we did not find support for a significant interaction between time and financial stress in predicting mental health (*β* = 0.019, *t*(628) = 0.77, *p* = 0.1 and *β* = 0.020, *t*(392) = 0.75, *p* = 1 for *t* = 0 and *t* = 1, respectively). This finding suggests that the strength of the relationship between financial stress and mental health did not change during the first six months of the COVID-19 pandemic. Of the control variables, only emotional stability explained mental health (*β* = −0.501, *t*(521) = −21.26, *p* < 0.001).

We did not find an association between time and financial stress (*β* = 0.0284, *t*(754) = 0.136, *p* = 0.737 and *β* = 0.052, *t*(500) = 2.46, *p* = 0.078 for *t* = 0 and *t* = 1, respectively). This finding indicates that, on average, financial stress during the first six months of COVID-19 did not differ from financial stress pre-COVID-19. Age was negatively associated with changes in financial stress (*β* = 0.0928, *t*(476) = −3.11, *p* = 0.013), indicating that financial stress levels of younger respondents increased during COVID-19. Also, we found that the group with the lowest education level (primary school) experienced more financial stress than the other groups. We did not find associations between gender (*β* = −0.067, *t*(637) = −2.73, *p* = 0.039) and household composition on the one hand and financial stress on the other. Of the three included personality traits, conscientiousness (*β* = −0.1188, *t*(415) = −4.37, *p* < 0.001) and emotional stability (*β* = −0.2403, *t*(517) = 8.84, *p* < 0.001) were negatively associated with financial stress increases.

In addition to the indirect relation (mediation) described above, we found that financial stress increases positively mediated the association between age (95% CI [0.00369, 0.02]), gender (95% CI [0.0025, 0.015]), and education level on the one hand and mental health decreases on the other (see Table 6). We found no support for an indirect association between household composition and mental health changes, with financial stress as the mediator. Finally, we found that financial stress increases also mediated the association between conscientiousness (95% CI [0.00666, 0.0232]) and emotional stability (95% CI [−0.0417, −0.0168]) on the one hand and mental health decreases on the other.

**Table 6:** Mediation analysis.

	Estimate	SE	95% CI	
Adjusted Income (*t* = 0)	−1.69e-02	1.09e-02	−4.00e-02	3.13e-03
Δ Adjusted income	2.08e-02	9.59e-03	3.71e-03	4.13e-02
Savings	1.68e-02	5.79e-03	6.62e-03	2.92e-02
Debts	−1.08e-02	3.91e-03	−1.92e-02	−3.96e-03
Age	1.10e-02	4.16e-03	3.69e-03	2.00e-02
Gender (F)	7.91e-03	3.32e-03	2.05e-03	1.50e-02
Education level: 2	5.77e-02	1.79e-02	2.65e-02	9.62e-02
Education level: 3	5.26e-02	1.82e-02	2.07e-02	9.19e-02
Education level: 4	5.49e-02	1.72e-02	2.48e-02	9.20e-02
Education level: 5	6.23e-02	1.81e-02	3.06e-02	1.01e-01
Education level: 6	7.14e-02	2.04e-02	3.55e-02	1.15e-01
Household: no partner, with children	−1.68e-02	1.64e-02	−5.09e-02	1.42e-02
Household: partner, no children	1.51e-02	7.90e-03	8.94e-04	3.19e-02
Household: partner, with children	−3.41e-03	8.09e-03	−1.98e-02	1.24e-02
Conscientiousness	1.41e-02	4.24e-03	6.66e-03	2.32e-02
Emotional stability	2.85e-02	6.34e-03	−4.17e-02	−1.68e-02
Extraversion	−9.03e-04	3.10e-03	−7.16e-03	5.18e-03

*SE* standard error, *95% CI* 95% confidence interval.

## Discussion

The current study examined the role of financial vulnerability and financial stress in explaining individual differences in mental health changes during COVID-19. In a longitudinal study, we compared mental health in a large sample of the Dutch population before and during the pandemic. We used a random intercepts model, which enabled us to analyze the dynamic relationships between financial stress and mental health. We operationalized mental health through the Mental Health Inventory (MHI-5)^60^, which asks respondents how often they felt nervous, down, calm, depressed, and happy in recent weeks. Financial stress is a psychological concept characterized by the subjective experience of lacking financial resources to cope with demands. We measured financial stress through the Psychological Inventory of Financial Scarcity (PIFS)^48,49^.

We found that changes in financial stress related negatively to changes in mental health during the pandemic. Having few liquid savings and having large amounts of consumer debt before the pandemic outbreak explained increased financial stress during the pandemic. Low levels of savings and high levels of consumer debt are two important aspects of financial vulnerability. Households with few savings are less protected against income shocks or unexpected expenditures. Especially in a time of economic uncertainty, lacking savings may result in feelings of not being in control of one’s financial situation and in worries about being unable to meet financial obligations. Thus, low savings levels may result in increased financial stress.

Similarly, having debts may trigger worries about being unable to repay them because of the anticipation of future income drops. Also, having debts may increase feelings of dependency on others^57^. Thus, having debts in economically uncertain times such as COVID-19 may increase financial stress. We also found that changes in financial stress mediated the relation between savings and debts on the one hand and changes in mental health on the other. Theoretically, the causal relationship between financial vulnerability and mental health could go in both directions. However, because we used savings and debts before the pandemic as independent variables, that does not seem likely in this case. The relationship could also be confounded by a variable we did not include in our model. Although we cannot make causal inferences, this finding confirms earlier findings that financial vulnerability may be a risk factor for mental health in a pandemic.

We found no support for income or income changes explaining financial stress changes. Savings and debts are better predictors of financial stress than having a low income. The finding that decreasing income does not explain increasing financial stress may be due to governments’ comprehensive income support packages immediately after the pandemic outbreak. As a result, few households experienced income drops during the third measurement. The variability in income may have been too small to explain variability in financial stress. We did not find support for an interaction between time and financial stress in predicting mental health, which suggests that the strength of the relationship between financial stress and mental health did not significantly change during the pandemic.

Mean levels of mental health did not change in the first six months of the pandemic compared to the pre-pandemic situation. This flat course of average mental health, however, masked underlying heterogeneity. For four out of five respondents, mental health either increased or decreased. This finding corroborates earlier findings of high proportions of unexplained heterogeneity in mental health development during COVID-19^8^.

Our results suggest that between-person differences in the changes in financial stress may partly explain the heterogeneity in changes in mental health after controlling for age, gender, education level, household composition, and personality traits. Our study adds to the fast-growing knowledge of mental health development during COVID-19. We had the opportunity to use longitudinal data collected before and during COVID-19. Earlier studies examining mental health during COVID-19 were mostly cross-sectional or utilized data collected during the pandemic only. To our knowledge, our study is the first to examine the role of pre-pandemic savings, debts, income, and financial stress in mental health changes during the pandemic.

There are also some limitations and opportunities for further research. First, we used data collected during the first year of the pandemic outbreak. The mental health consequences of the COVID-19 crisis may be present for a long time and peak later than the actual pandemic^4^. Also, there is ample evidence of the effects of chronic stress on physical and mental health and childhood development^80,81^. For these reasons, it may be fruitful to extend the study of mental health development and (financial) stress to include more prolonged periods. Second, we examined the role of financial stress in general mental health changes during COVID-19. Future studies could examine the role of financial stress during COVID-19 for a broader range of mental health symptoms and disorders, such as post-traumatic stress, insomnia, and loneliness. A third avenue for further research lies in understanding the effect of financial stress on physical health development. There is rich literature on the relationship between socio-economic status and aspects of physical health, such as cardiovascular disease, arthritis, diabetes, chronic respiratory diseases, and cervical cancer^50,51^. Examining the prolonged effects of financial stress during COVID-19 in developing these and other illnesses would be worthwhile. Such examinations could help disentangle the complex relationship between socio-economic status and health and the role of lifestyle therein. They could establish the relative contribution of the different pathways (i.e., through the disease itself, the pandemic containment measures, and the economic consequences of the pandemic).

The results of our study point to several policy implications. First, our results confirm the importance of safeguarding financial security for financially vulnerable households in crises. Soon after the outbreak, governments worldwide implemented unprecedented income support packages. These support packages are currently being phased out while economic consequences may endure or only start to arise. Financially vulnerable households are the most likely to experience the prolonged economic consequences of the pandemic in the aftermath of the health crisis because they do not have the financial resources to deal with economic shocks.

Second, mental health programs should include financially vulnerable groups. Many of the studies referenced in this article have called upon health professionals, policymakers, and researchers to develop interventions to counter the adverse psychological consequences of the pandemic, especially for vulnerable groups^3,7,21^. The current study results confirm that such programs should reach out to financially vulnerable households and address their specific mental health needs.

Third, mental health interventions should address the psychological symptoms of COVID-19, such as post-traumatic stress, anxiety, depression, loneliness, and insomnia, and prevent such symptoms by mitigating financial stress because control is an essential aspect of financial stress. Financial counseling and coaching to increase control and self-efficacy provide promising avenues for reducing financial stress and promoting mental health, especially for financially vulnerable households^48,82^.

Finally, an important lesson for future pandemics and other economic shocks is promoting buffer savings and avoiding unnecessary debts. This may make households more resilient to the adverse mental health consequences of future shocks. In sum, policymakers and professionals from mental health and finance can benefit from the notion that mental health and financial security go hand in hand by incorporating financial security into mental health programs and vice versa.

### Supplementary Information

Supplemental materials

Reporting Summary

## Data Availability

The current study used data from the LISS panel administered by CentERData^58^. Researchers are encouraged to contact CentERData to obtain the datasets used in this study. Detailed instructions for accessing LISS panel data are available here: https://www.lissdata.nl/access-data. A list of data sets used in the current study is available at the Open Science Framework (OSF): https://osf.io/4ctsr/. CentERDAta policy does not allow authors to provide access to data sets directly to other researchers.

## Code availability

We used R version 4.1.0^83^ to perform statistical analyses. The code used to process that data and perform the analyses are available from the Open Science Framework through the following link: https://osf.io/4ctsr/.

## References

[1] World Health Organization. *WHO Director-General’s opening remarks at the media briefing on COVID-19 - 11 March 2020*. https://www.who.int/director-general/speeches/detail/who-director-general-s-opening-remarks-at-the-media-briefing-on-covid-19---11-march-2020 (2020).

[2] Cullen W, Gulati G, Kelly BD (2020). Mental health in the COVID-19 pandemic. QJM Int. J. Med..

[3] World Health Organization. *Mental Health And Psychosocial Considerations During The Covid-19 Outbreak* (World Healt Organization, 2019).

[4] Sher L (2020). The impact of the COVID-19 pandemic on suicide rates. QJM Int. J. Med..

[5] Talevi D (2020). Mental health outcomes of the CoViD-19 pandemic. Riv. Psichiatr..

[6] Tsai J, Elbogen EB, Huang M, North CS, Pietrzak RH (2021). Psychological distress and alcohol use disorder during the COVID-19 era among middle- and low-income U.S. adults. J. Affect. Disord..

[7] Rogers JP (2020). Psychiatric and neuropsychiatric presentations associated with severe coronavirus infections: a systematic review and meta-analysis with comparison to the COVID-19 pandemic. Lancet Psychiatry.

[8] Robinson E, Sutin AR, Daly M, Jones A (2022). A systematic review and meta-analysis of longitudinal cohort studies comparing mental health before versus during the COVID-19 pandemic in 2020. J. Affect. Disord..

[9] Wang C (2020). A longitudinal study on the mental health of general population during the COVID-19 epidemic in China. Brain Behav. Immun..

[10] Wang, C. et al. Immediate psychological responses and associated factors during the initial stage of the 2019 coronavirus disease (COVID-19) epidemic among the general population in China. *Int. J. Environ. Res. Public Health* **17**, 1729 (2020). 10.3390/ijerph17051729 PMC7084952 32155789

[11] O’Connor RC (2021). Mental health and wellbeing during the COVID-19 pandemic: Longitudinal analyses of adults in the UK COVID-19 Mental Health & Wellbeing study. Br. J. Psychiatry.

[12] Every-Palmer, S. et al. Psychological distress, anxiety, family violence, suicidality, and wellbeing in New Zealand during the COVID-19 lockdown: a cross-sectional study. *PLoS ONE* **15**, e0241658 (2020). 10.1371/journal.pone.0241658 PMC7641386 33147259

[13] Dutch Social Planning Office. *One Year With COVID-19.* https://www.scp.nl/publicaties/publicaties/2021/03/03/een-jaar-met-corona (2021).

[14] Pirkis J (2021). Suicide trends in the early months of the COVID-19 pandemic: An interrupted time-series analysis of preliminary data from 21 countries. Lancet Psychiatry.

[15] Kumar A, Nayar KR (2021). COVID 19 and its mental health consequences. J. Mental Health.

[16] Brooks SK (2020). The psychological impact of quarantine and how to reduce it: Rapid review of the evidence. The Lancet.

[17] Pfefferbaum B, North CS (2020). Mental health and the COVID-19 pandemic. New Engl. J. Med..

[18] De Pue S (2021). The impact of the COVID-19 pandemic on wellbeing and cognitive functioning of older adults. Sci. Rep..

[19] Groarke JM (2020). Loneliness in the UK during the COVID-19 pandemic: Cross-sectional results from the COVID-19 Psychological Wellbeing Study. PLoS ONE.

[20] Ellwardt L, Präg P (2021). Heterogeneous mental health development during the COVID-19 pandemic in the United Kingdom. Sci. Rep..

[21] Holmes EA (2020). Multidisciplinary research priorities for the COVID-19 pandemic: A call for action for mental health science. Lancet Psychiatry.

[22] Armour C, McGlinchey E, Butter S, McAloney-Kocaman K, McPherson KE (2021). The covid-19 psychological wellbeing study: understanding the longitudinal psychosocial impact of the covid-19 pandemic in the uk; a methodological overview paper. J. Psychopathol. Behav. Assess..

[23] Usher K, Durkin J, Bhullar N (2020). The COVID‐19 pandemic and mental health impacts. Int. J. Ment. Health Nurs..

[24] Mental Health Foundation. The COVID-19 pandemic, financial inequality and mental health. *Mental Health Foundation* https://www.mentalhealth.org.uk/our-work/research/coronavirus-mental-health-pandemic/covid-19-inequality-briefing (2020).

[25] Hamilton R (2021). Scarcity and coronavirus. J. Public Policy Mark..

[26] Torres O (2022). Risk of burnout in French entrepreneurs during the COVID-19 crisis. Small Bus. Econ..

[27] Kanter JB, Williams DT, Rauer AJ (2021). Strengthening lower-income families: Lessons learned from policy responses to the COVID-19 pandemic. Family Process.

[28] Sugawara, D., Masuyama, A. & Kubo, T. Socioeconomic impacts of the COVID-19 lockdown on the mental health and life satisfaction of the japanese population. *Int. J. Ment. Health Addict.*10.1007/s11469-020-00461-3 (2021). 10.1007/s11469-020-00461-3 PMC8638648 34876889

[29] Wu, X., Li, X., Lu, Y. & Hout, M. Two tales of one city: unequal vulnerability and resilience to COVID-19 by socioeconomic status in Wuhan, China. *Res. Soc. Stratif. Mobil.* **72**, 100584 (2021). 10.1016/j.rssm.2021.100584 PMC7881731 33612911

[30] Kantamneni, N. The impact of the COVID-19 pandemic on marginalized populations in the United States: a research agenda. *J. Vocat. Behav.* **119**, 103439 (2020). 10.1016/j.jvb.2020.103439 PMC7205696 32390658

[31] Patel JA (2020). Poverty, inequality and COVID-19: the forgotten vulnerable. Public Health.

[32] Eurofound. *Living, Working and COVID-19*. https://www.eurofound.europa.eu/publications/report/2020/living-working-and-covid-19 (2020).

[33] Ettman CK (2021). Low assets and financial stressors associated with higher depression during COVID-19 in a nationally representative sample of US adults. J. Epidemiol. Community Health.

[34] Reme, B.-A., Wörn, J. & Skirbekk, V. Longitudinal evidence on the development of socioeconomic inequalities in mental health due to the COVID-19 pandemic in Norway. *Sci. Rep.* **12**, 3837 (2022). 10.1038/s41598-022-06616-7 PMC8907231 35264610

[35] Zhou M, Guo W (2021). Subjective distress about COVID-19 and its social correlates: Empirical evidence from hubei province of china. J. Affect. Disord..

[36] Pijpker R, van der Kamp D, Vader S, den Broeder L, Wagemakers A (2022). Socioeconomic status and mental health during the COVID-19 crisis: Are sense of coherence, sense of community coherence and sense of national coherence predictors for mental health?. Health Psychol. Rep..

[37] Pereira I, Patel PC (2022). Impact of the COVID-19 pandemic on the hours lost by self-employed racial minorities: evidence from Brazil. Small Bus. Econ..

[38] Kalenkoski CM, Pabilonia SW (2022). Impacts of COVID-19 on the self-employed. Small Bus. Econ..

[39] Vinberg, S., Landstad, B. J., Tjulin, A. & Nordenmark, M. Sickness presenteeism among the Swedish self-employed during the COVID-x19 pandemic. *Front. Psychology* **12**, 723036 (2021). 10.3389/fpsyg.2021.723036 PMC8490737 34621220

[40] Xu, Z. & Jia, H. The influence of COVID-19 on entrepreneurs’ psychological wellbeing. *Front. Psychol.* **12**, 823542 (2022). 10.3389/fpsyg.2021.823542 PMC8795827 35095701

[41] Willeke K (2021). Occurrence of mental illness and mental health risks among the self-employed: a systematic review. Int. J. Environ. Res. Public Health.

[42] Rose G, Marmot MG (1981). Social class and coronary heart disease. Heart.

[43] Adler NE (1994). Socioeconomic status and health: the challenge of the gradient. Am. Psychol..

[44] Richardson T, Elliott P, Roberts R (2013). The relationship between personal unsecured debt and mental and physical health: a systematic review and meta-analysis. Clin. Psychol. Rev..

[45] Ridley, M., Rao, G., Schilbach, F. & Patel, V. Poverty, depression, and anxiety: causal evidence and mechanisms. *Natl Bur. Econ. Res.*10.3386/w27157 (2020). 10.1126/science.aay0214 33303583

[46] Mullainathan, S. & Shafir, E. *Scarcity: Why Having Too Little Means So Much* (Times Books, Macmillan and Henry Holt, 2013).

[47] Shah AK, Mullainathan S, Shafir E (2012). Some consequences of having too little. Science.

[48] Van Dijk, W. W., Van der Werf, M. M. B., & Van Dillen, L. F. The psychological inventory of financial scarcity (PIFS): a psychometric evaluation. *J. Behav. Exp. Econ.* **101**, 101939 (2022).

[49] Hilbert, L. P., Noordewier, M. K. & van Dijk, W. W. The prospective associations between financial scarcity and financial avoidance. *J. Econ. Psychol.* **88**, 102459 (2022).

[50] Cundiff, J. M., Wicherts, J. M. & Muscatell, K. A. The pathway from social status to physical health: Taking a closer look at stress as a mediator. *Curr. Direct. Psychol. Sci.*10.1177/0963721420901596 (2020).

[51] Adler NE, Snibbe AC (2003). The role of psychosocial processes in explaining the gradient between socioeconomic status and health. Curr. Direct. Psychol. Sci..

[52] McLeod, J. D. & Kessler, R. C. Socioeconomic status differences in vulnerability to undesirable life events. *J. Health Soc. Behav.* **31**, 162–172 (1990). 2102495

[53] Drentea P (2000). Age, debt and anxiety. J. Health Soc. Behav..

[54] Hannagan, A. & Morduch, J. Income gains and month-to-month income volatility: Household evidence from the us financial diaries. NYU Wagner Research Paper No. 2659883, US Financial Diaries Working Paper, 2015, *SSRN Journal*10.2139/ssrn.2659883 (2015).

[55] Prause J, Dooley D, Huh J (2009). Income volatility and psychological depression. Am. J. Community Psychol..

[56] Ruberton PM, Gladstone J, Lyubomirsky S (2016). How your bank balance buys happiness: The importance of ‘cash on hand’ to life satisfaction. Emotion.

[57] Drentea, P. & Reynolds, J. R. Where does debt fit in the stress process model? *Soc. Ment. Health*. **5**, 16–32 (2015). 10.1177/2156869314554486 PMC6521877 31106006

[58] Scherpenzeel, A. & Das, M. *Social and Behavioral Research and the Internet: Advances in Applied Methods and Research Strategies*. in (eds. Das, M., P. Ester, and L. Kaczmirek). p. 77-104 (Routledge, 2010).

[59] Rumpf H-J, Meyer C, Hapke U, John U (2001). Screening for mental health: validity of the MHI-5 using DSM-IV Axis I psychiatric disorders as gold standard. Psychiatry Res..

[60] Ware, J., Snoww, K., MA, K. & BG, G. Sf36 health survey: manual and interpretation guide. Vol. 30 (Quality Metric, Inc, 1993).

[61] OECD. *OECD Framework for Statistics on the Distribution of Household Income, Consumption and Wealth*. 10.1787/9789264194830-en (2013).

[62] Goldberg LR (1992). The development of markers for the Big-Five factor structure. Psychol. Assess..

[63] Brown AL, Lahey JN (2015). Small victories: creating intrinsic motivation in task completion and debt repayment. J. Mark. Res..

[64] Donnelly G, Iyer R, Howell RT (2012). The Big Five personality traits, material values, and financial wellbeing of self-described money managers. J. Econ. Psychol..

[65] Streiner DL (2015). Best (but oft-forgotten) practices: the multiple problems of multiplicity—whether and how to correct for many statistical tests. Am. J. Clin. Nutr..

[66] Benjamini Y, Hochberg Y (1995). Controlling the false discovery rate: a practical and powerful approach to multiple testing. J. R. Stat. Soc. Ser. B (Methodol.).

[67] Van Buuren, S. *Flexible Imputation Of Missing Data* (CRC press, 2018).

[68] Asendorpf JB, Van De Schoot R, Denissen JJ, Hutteman R (2014). Reducing bias due to systematic attrition in longitudinal studies: the benefits of multiple imputation. Int. J. Behav. Dev..

[69] Quartagno, M., Grund, S. & Carpenter, J. Jomo: a flexible package for two-level joint modelling multiple imputation. *R Journal* **9** (2019).

[70] Allison, P. *Fixed Effects Regression Models*. 10.4135/9781412993869 (2009).

[71] Huque MH, Carlin JB, Simpson JA, Lee KJ (2018). A comparison of multiple imputation methods for missing data in longitudinal studies. BMC Med. Res. Methodol..

[72] Satterthwaite FE (1946). An approximate distribution of estimates of variance components. Biometrics.

[73] Von Hippel, P. T. How many imputations do you need? A two-stage calculation using a quadratic rule. *SAGE J*. 10.1177/0049124117747303 (2020). 10.1177/0049124117747303 PMC11361408 39211325

[74] Rubin, D. B. *Multiple Imputation For Survey Nonresponse* (Wiley, 1987).

[75] Koller M (2016). Robustlmm: an R package for robust estimation of linear mixed-effects models. J. Stat. Softw..

[76] Wilcox, R. R. *Introduction To Robust Estimation And Hypothesis Testing* (Academic Press, 2012).

[77] Roth, D. L. & MacKinnon, D. P. *Longitudinal Data Analysis: A Practical Guide For Researchers In Aging, Health, And Social Sciences.* p. 181–216 (Routledge/Taylor & Francis Group, 2012).

[78] Tofighi D, MacKinnon DP (2011). RMediation: an R package for mediation analysis confidence intervals. Behav. Res. Methods.

[79] Health Council. *Kernadvies Mentale gevolgen van de coronapandemie: een eerste inventarisatie [Advice Mental consequences of the corona pandemic: a first inventorisation]*. (2022).

[80] McEwen BS (2012). Brain on stress: How the social environment gets under the skin. Proc. Natl Acad. Sci. USA.

[81] Lupien SJ, McEwen BS, Gunnar MR, Heim C (2009). Effects of stress throughout the lifespan on the brain, behaviour and cognition. Nat. Rev. Neurosci..

[82] White ND, Packard K, Kalkowski J (2019). Financial education and coaching: a lifestyle medicine approach to addressing financial stress. Am. J. Lifestyle Med..

[83] R Core Team. *R: A Language and Environment for Statistical Computing*. (R Foundation for Statistical Computing, 2020).

