# Supplementary material for: The role of financial stress in mental health changes during COVID-19
Source: Npj Ment Health Res. 2022 Oct 14;1:15. doi: 10.1038/s44184-022-00016-5 (PMC9568931; doi:10.1038/s44184-022-00016-5)
Supplement: Supplementary file 1 — Supplemental materials [file 44184_2022_16_MOESM1_ESM.pdf]

The role of financial stress in mental health trajectories during COVID-19

Supplemental materials

Supplementary Table 1. Sample characteristics by mental health change

Characteristic	Decreased, N = 3341	Unchanged, N = 1781	Increased, N = 3211
Net Income (t=0)	32,400 (22,068, 46,755)	30,648 (23,441, 42,723)	34,417 (21,025, 46,080)
Net Income (*t = 0*)	32,950 (22,460, 47,075)	33,290 (25,200, 46,350)	35,280 (22,482, 49,245)
Net Income (*t* = 1)	33,480 (22,695, 48,796)	34,186 (25,200, 47,020)	35,868 (22,800, 50,400)
Adjusted Income (*t* = 0)	21,912 (16,795, 29,422)	23,011 (17,770, 28,710)	21,949 (16,981, 29,556)
Adjusted Income (*t = 1*)	22,708 (17,518, 30,043)	25,329 (19,092, 31,200)	24,529 (17,521, 32,502)
Adjusted Income (*t* = 2)	23,331 (17,395, 31,291)	25,584 (19,092, 32,117)	24,549 (17,395, 33,213)
Age	54.2 (17.4) [18.0 91.0]	57.1 (15.0) [20.0 87.0]	53.1 (17.3) [18.0 90.0]
Education Level			
1: primary school	19 (5.7%)	9 (5.1%)	18 (5.6%)
2: vmbo (intermediate secondary education)	69 (21%)	42 (24%)	64 (20%)
3: havo/vwo (higher secondary education)	42 (13%)	15 (8.4%)	40 (12%)
4: mbo (intermediate vocational education)	84 (25%)	37 (21%)	85 (26%)
5: hbo (higher vocational education)	88 (26%)	56 (31%)	74 (23%)
6: wo (university)	31 (9.3%)	19 (11%)	40 (12%)
Gender: Female	183 (55%)	84 (47%)	177 (55%)
Household Composition			
1: no partner, no children	97 (29%)	56 (31%)	93 (29%)
2: no partner, with children	17 (5.1%)	7 (3.9%)	10 (3.1%)
3: partner, no children	130 (39%)	72 (40%)	129 (40%)
4: partner, with children	90 (27%)	43 (24%)	89 (28%)
Buffer? (Y)	122 (68%)	70 (75%)	123 (73%)
No. Debts			
0	287 (89%)	162 (95%)	277 (89%)

Characteristic	Decreased, N = 3341	Unchanged, N = 1781	Increased, N = 3211
1	35 (11%)	8 (4.7%)	31 (10.0%)
2	1 (0.3%)	1 (0.6%)	3 (1.0%)
5	1 (0.3%)	0 (0%)	0 (0%)
Financial Stress (t=0)	1.79 (1.07) [0.92 6.42]	1.55 (0.91) [0.92 4.75]	1.80 (0.97) [0.92 5.25]
Financial Stress (*t = 0*)	1.81 (1.06) [0.92 6.17]	1.51 (0.84) [0.92 5.00]	1.81 (1.04) [0.92 5.92]
Financial Stress (t=2)	1.67 (1.01) [0.92 6.42]	1.41 (0.78) [0.92 4.25]	1.66 (0.93) [0.92 5.75]
Mental Health Index (t=0)	4.31 (0.69) [1.40 5.40]	4.52 (0.78) [1.20 5.40]	3.77 (0.91) [1.00 5.20]
Mental Health Index (*t = 0*)	4.04 (0.80) [0.60 5.40]	4.42 (0.86) [0.40 5.40]	4.03 (0.86) [1.20 5.40]
Mental Health Index (t=2)	3.75 (0.83) [0.40 5.20]	4.52 (0.78) [1.20 5.40]	4.35 (0.72) [1.40 5.40]
